# Establishment of cohesion 1 homolog 2 facilitates cell aggressive behaviors and induces poor prognosis in renal cell carcinoma

**DOI:** 10.1002/jcla.23163

**Published:** 2020-01-15

**Authors:** Qiu‐Li Wang, Ling Liu

**Affiliations:** ^1^ Department of Nephrology Jining NO.1 People's Hospital Shandong China

**Keywords:** cell behaviors, ESCO2, p53, PCNA, renal cell carcinoma

## Abstract

**Background and aims:**

Establishment of cohesion 1 homolog 2 (ESCO2) has been identified as an essential factor for cohesion in cell cycle in human multiple cancers. Nonetheless, its functional implication on prognosis and cellular behaviors of renal cell carcinoma (RCC) is rarely elucidated. We performed this study to detect the effects of ESCO2 in RCC progression.

**Methods:**

We accessed The Cancer Genome Atlas (TCGA) database to evaluate the ESCO2 expression levels in tumor tissues, including 32 normal tissues and 289 tumor tissues. Quantitative real‐time PCR and Western blot were implemented for expression detection. After ESCO2 knockdown using siRNAs interference, functional experiments were conducted to explore the role of ESCO2, such as cell proliferation analysis and colony formation assay. Transwell assays for migration and invasion was also performed.

**Results:**

In this study, ESCO2 was significantly increased in RCC tissues and cell lines. The RCC patients with high expression of ESCO2 were susceptible to unfavorable prognosis, and its expression has a marked association with clinical features containing age, gender, pathologic stage, and so on. Furthermore, knockdown of ESCO2 inhibited cell growth, invasion, and migration. Mechanistically, phosphorylation protein kinase B (AKT) and mammalian target of rapamycin (mTOR), proliferating cell nuclear antigen (PCNA), and p53 were all down‐regulated due to the ESCO2 inhibition.

**Conclusions:**

Therefore, our results raised the possibility that ESCO2 may act as a promising option for tumor therapeutic interference by exhibiting enhanced selectivity over conventional chemotherapy.

## 1 INTRODUCTION

Kidney cancer ranks second in urogenital tumors in the world with an increasing incidence and mortality rate of 2.2% and 1.8% in 2018, respectively.^1^ Renal cell carcinoma (RCC) is one of the most common kidney cancer, which can be divided into four subtypes including clear cell RCC, papillary RCC, renal oncocytoma, and chromophobe RCC.^2^ The current therapeutic therapies for RCC are mainly surgical resection, chemotherapy, and radiotherapy. However, the response to the mentioned treatments is un‐effective.^3^ Thus, we hope that the great progress in targeted treatments would emerge with the fully understanding of RCC pathogenesis in the future.

Establishment of cohesion 1 homolog 2 (ESCO2) is a member of Eco1 family, which contributes to sister chromatid cohesion (SCC) during cell cycle progression.^4^ It is well‐known that distinct statuses of cohesin are associated with different phases of cell cycle.^5^ Roberts syndrome (RBS) has ascertained correlation with the inactivation of ESCO2, which is caused by the SCC defects and fallacious transcription of acetylation.^6^, ^7^ Furthermore, SCC is responsible for DNA repair in cell proliferation.^8^ ESCO2 that holds the regulatory role is involved in the cohesion‐mediated DNA repair. Due to the above mentioned points, more and more attentions have paid to the biological functions of ESCO2 in cancer development. Automatically, increasing investigations have indicated the significance of ESCO2 in tumorigenesis. Fumiichiro and Miyako examined the genes change, found that up‐regulation of ESCO2 was striking in breast cancer tissues and cell lines.^9^ Notably, the dysregulated ESCO2 was implicated to adjust metastatic activity in colorectal cancer (CRC), suppressing Matrix Metallopeptidase 2 (MMP2).^10^ Yet, whether and how the progression of RCC is controlled by ESCO2 has not been resolved.

Here, we performed this study to assess the expression of ESCO2 in RCC tissues and cell lines, investigated the association between ESCO2 and prognosis of patients with RCC and detected the biological role of ESCO2 knockdown on the cellular malignant behaviors in vitro. Additionally, the potential mechanism underlying the impact of ESCO2 in RCC progression was also explored using Western blot analysis.

## 2 MATERIALS AND METHODS

### 2.1 Data collection

The RCC gene expression profiles and clinical data containing 32 normal specimens and 289 tumor specimens were collected from TCGA database.

### 2.2 Cell culture and transfection

Human tubular epithelial cells HK2 and renal cancer cell lines (786‐O, KETR3, G401) were obtained from the Cell Biology of the Chinese Academy of Sciences (Shanghai, China). All the cell lines were incubated in Roswell Park Memorial Institute‐1640 (RPMI‐1640) medium, supplemented with 10% fetal bovine serum, 1% penicillin and streptomycin at 37°C in a humidified 5% CO_2_ atmosphere.

Subsequently, cell transfection was conducted on the basis of the manufacturer's instructions by the Lipofectamine2000 (Invitrogen). To inhibit the expression of ESCO2 gene, the relevant siRNAs molecules including the negative control siRNA were designed and synthesized by RiboBio Co Ltd. The sequences are exhibited as follows: si‐ESCO2: 5′‐CACTCTTAGACCAGGATTATC‐3′; si‐con: 5′‐AATTCTCCGAACGTGTCACGT‐3′.

### 2.3 Quantitative real‐time PCR analysis

Total RNA was isolated from the cultured cells utilizing TRIzol solution in accordance with the manufacturer's protocol, and next, RNA was reverse transcribed into cDNA with the help of the iScript cDNA Synthesis Kit (Bio‐rad). For detection of the ESCO2 expression level, real‐time PCR were performed relying on the subsequent procedure: 95°C × 5 minutes, 95°C × 30 seconds, 60°C × 45 seconds with 40 cycles, and 72˚C × 30 seconds. The indicated primers were presented as follows:

ESCO2: F, 5′‐TGGGATAAGTAGAATCTGGGTT‐3′

R, 5′‐ATACGAGGAAATTAGGGGTGT‐3′

GAPDH: F, 5′‐ACACCCACTCCTCCACCTTT −3′

R, 5′‐TTACTCCTTGGAGGCCATGT −3′

The expression level of ESCO2 was normalized to GAPDH, which served as the internal control, and 2^‐ΔΔCt^ method was used to analyze the data. Each experiment was repeated in three triplicates.

### 2.4 Western blotting

Protein lysates were collected from cells that treated by si‐ESCO2 or si‐con, and 1 mmol/L phenylmethylsulfonyl fluoride (PMSF; protease inhibitor) was added into RIPA buffer to extract protein successfully. Then, protein was separated in 10% sodium dodecyl sulfate‐polyacrylamide gel electrophoresis (SDS‐PAGE) and transferred to polyvinylidene difluoride (PVDF) membranes. Finally, the target proteins were probed using primary antibodies and secondary antibodies. All the antibodies including ESCO2 (ab86003), GAPDH (ab181602), AKT (ab179463), p‐AKT (ab38449), mTOR (ab134903), p‐mTOR (ab109268), PCNA (ab92552), and p53 (ab32389) were purchased from Abcam. The protein bands were developed by enhanced chemiluminescence reagent (Thermo Fisher Scientific), and QUANTITY ONE was applied to detect the gray values of proteins.

### 2.5 CCK‐8 and colony formation assay

After 24 hours transfection, CCK‐8 assay was performed to determine the cell proliferative potential at 0, 24, 48, and 72 hours on the basis of standard instructions. And the optical density (OD) value was measured at wavelength of 450 nm under a microplate reader.

Cells with siRNAs interference were cultured at 37°C with 5% CO_2_ for about 2 weeks, and then, the visible colonies were fixed in 4% paraformaldehyde, stained with 0.1% crystal violet, imaged and calculated under a microscope. Triplicate independent experiments were conducted.

### 2.6 Cell invasion and migration assay

Cell invasion and migration were examined as above described after 48 hours transfection. For cell invasion assay, transwell chamber with 8‐μm pore size and Matrigel (BD Biosciences) were employed. The upper chamber was filled with 100 μL of cell suspension that contains 1 × 10^5^ cells, meanwhile, the bottom chamber was covered with 500 μL serum‐free medium. After overnight, non‐invasive cells were wiped out from the top chamber using a cotton swab. Subsequently, invasive cells were washed by PBS, fixed in paraformaldehyde and dyed in crystal violet. Using a microscope, five fields were selected randomly and the number of invasive cells was counted and averaged.

For cell migration assay, the density of inoculated cells was 5 × 10^3^ and the chamber does not need to be coated with Matrigel. Other than that, the other steps are the same as cell invasion assay.

### 2.7 Statistical analysis

Statistical analyses were performed through the SPSS 22.0 software and GraphPad Prism 5.0. Comparisons in two groups were analyzed by chi‐square test, and the differences in three or more groups were determined through one‐way ANOVA with Dunnett's post hoc test. The survival analysis was detected via Kaplan‐Meier method and log‐rank test, and Cox regression analysis was enforced for the prediction of prognostic factors. All the data were presented as mean ± standard deviation (SD). *P* < .05 was defined as statistical importance.

## 3 RESULTS

### 3.1 ESCO2 expression was significantly increased in RCC tissue samples and cell lines

**Figure 1:**
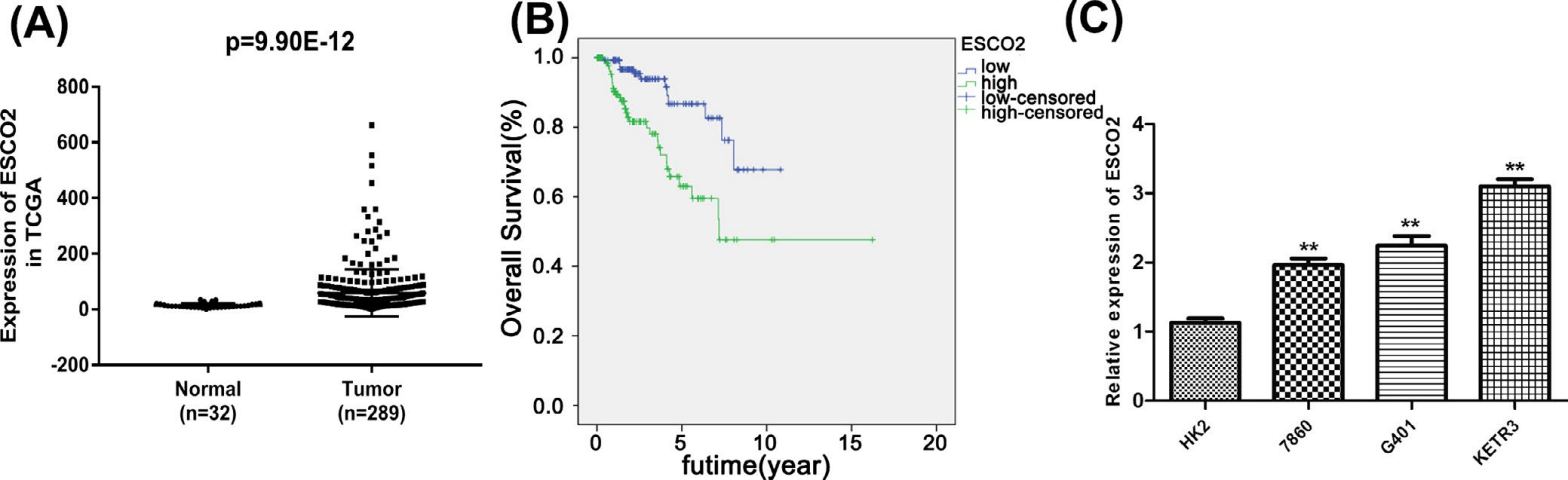
Establishment of cohesion 1 homolog 2 expression was elevated and induced poor prognosis in RCC. A, The comparison of ESCO2 expression in RCC tissues and human normal tissues, *P* = 9.90E‐12. B, The short survival period was related with the over‐expression of ESCO2, *P* < .01. C, Relative expression of ESCO2 in RCC cell lines, **P < .01 compared with si‐con group

To determine whether there is a difference in the expression level of ESCO2 between normal tissues and tumor tissues, 32 normal cases and 289 tumor cases from TCGA database were used in this present study. As a result, ESCO2 expression in RCC tissues was higher than that in normal controls (Figure 1A, *P* < .05). In addition, we assessed the endogenous expression of ESCO2 in several cell lines including HK2, 786‐O, KETR3, and G401. According to qRT‐PCR analysis, the expressional pattern were diverse although the ESCO2 was highly regulated in three tumor cell lines compared with the human tubular epithelial cells HK2 cells (Figure 1C, *P* < .01). Due to the ESCO2 expression in KETR3 cells ranking the top, thus, we selected the KETR3 cell line as the material in future experiments.

### 3.2 High‐regulation of ESCO2 was associated with unfavorable prognosis in RCC

To further examine the association between expression level of ESCO2 and progression of RCC, we collected and analyzed the clinical data. From the Figure 1B, we observed that the RCC patients with high ESCO2 expression suffered poor outcomes (*P* < .01). Moreover, there was a certain correlation between the expression level of ESCO2 and clinical features (Table 1, *P* < .05). These data showed that ESCO2 expression was related with Age (*P* = .014), Gender (*P* = .007), Pathologic‐Stage (*P* = .000), Pathologic‐T (*P* = .000) and Pathologic‐N (*P* = .000). Hence, we speculated that ESCO2 may be a novel regulator in the development of RCC.

**Table 1:** Association between ESCO2 and the clinicopathological characteristics of RCC patients based on the TCGA database

Characteristics	Expression of ESCO2		*P* value
	Low	High	
Age			
<60	49	69	.014*
≥60	93	72	
Gender			
Female	28	48	.007*
Male	115	94	
Pathologic‐stage			
I + II	113	78	.000*
III + IV	13	52	
Pathologic‐T			
T1 + T2	131	92	.000*
T3 + T4	11	49	
Pathologic‐N			
N0	24	25	.000*
N1	1	26	
Pathologic‐M			
M0	42	53	.054
M1	1	8	

Abbreviations: M, metastasis; N, lymph nodes; T, tumor.

* *P* < .05.

### 3.3 Knockdown of ESCO2 undermined cancer‐related cellular malignant behaviors

**Figure 2:**
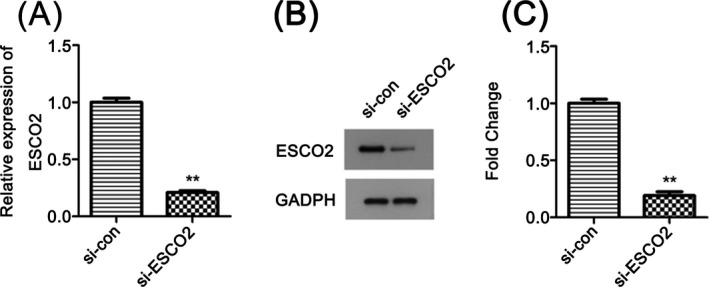
Transfection efficiency of si‐ESCO2. A and B, si‐ESCO2 knockdown the expression of ESCO2 effectively, quantified in C, **P < .01 compared with si‐con group

Next, we used si‐ESCO2 to transfect KETR3 cells and then obstructed the expression of ESCO2. The results showed that ESCO2 was excluded successfully by qRT‐PCR method and Western blot analysis (Figure 2A‐C, *P* < .01).

**Figure 3:**
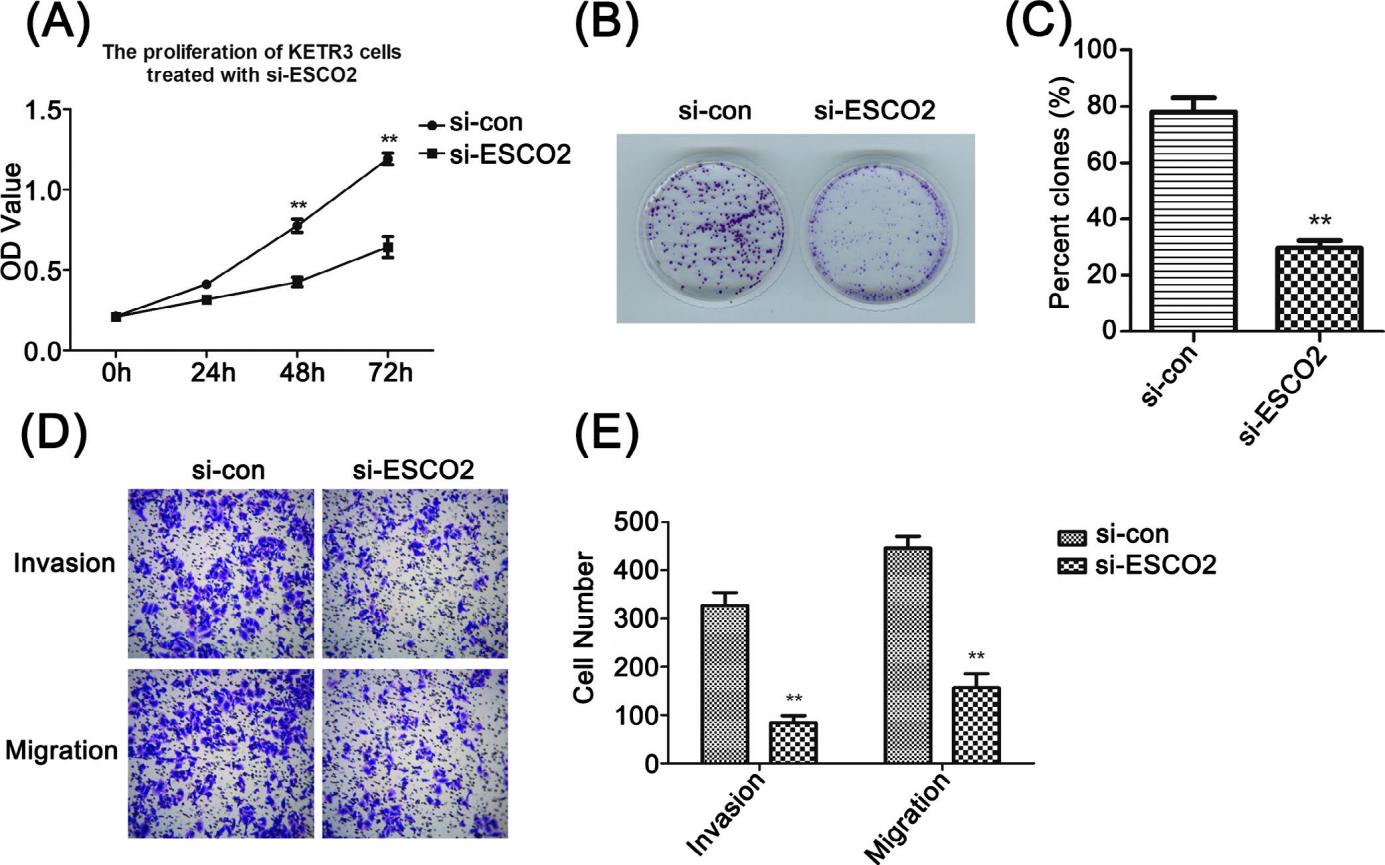
Knockdown of ESCO2 impaired cell proliferation, invasion, and migration in RCC. A, CCK8 analysis of cell viability in ESCO2‐knockdown KETR3 cells at 0, 24, 48, and 72 h, respectively, **P < .01 compared with si‐con group. B, ESCO2 silencing repressed colony formation. C, The colony‐forming rate was analyzed as the following equation: (colony number/seeded cell number) × 100%, **P < .01 compared with si‐con group. D, Cell invasion and migration were measured using transwell assay, and the migratory or invasive cells were calculated in E, **P < .01 compared with si‐con group

Based on ESCO2 knockdown cell model, we performed biological experiments to elevate the role of ESCO2 on malignant cellular behaviors. CCK‐8 assay indicated that reduction of ESCO2 hindered cell viability compared with the control (Figure 3A, *P* < .01), simultaneously, the impairment of clone potential verified the effect of ESCO2 through colony formation assay (Figure 3B, 3, *P* < .01). Furthermore, transwell analysis revealed that knockdown of ESCO2 markedly suppressed cell migration and invasion (Figure 3D, 3, *P* < .01).

### 3.4 Down‐regulation of ESCO2 was related with the inactivation of the AKT/mTOR pathway in RCC

**Figure 4:**
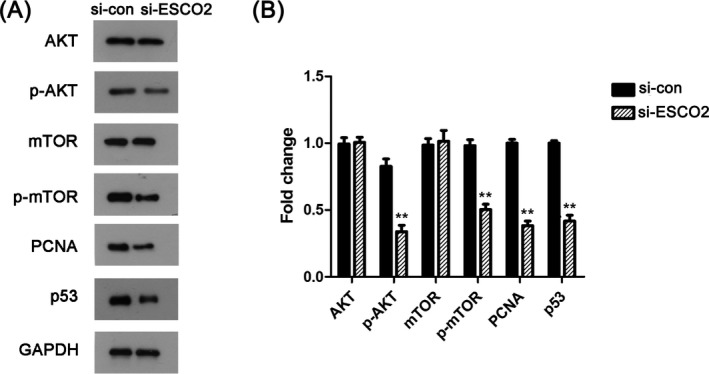
The regulation of ESCO2 in RCC was associated with the AKT/mTOR pathway. A, Western blot results manifested the down‐regulated expression of p‐AKT, p‐mTOR, PCNA, and p53. B, The relative expression was quantified, **P < .01 compared with si‐con group

Hereafter, on the basis of above results, we used Western blotting to investigate the expression level of the AKT/mTOR signaling pathway‐related proteins, which included AKT, p‐AKT, mTOR, and p‐mTOR. After knockdown ESCO2, we found that the expressions of p‐AKT and p‐mTOR were significantly decreased. On the contrary, there were no obvious differences in the expression of AKT and mTOR (Figure 4A). In addition to these, PCNA and p53 as important hallmarks involved in cell proliferation were also detected. Compared with the si‐con group, PCNA and p53 were remarkably declined. As shown in Figure 4B, the mentioned results were verified by quantified in bar chart (*P* < .05). In general, the AKT/mTOR pathway may be an important signaling pathway involved in the regulation of ESCO2 in RCC.

## 4 DISCUSSION

Renal cell carcinoma is well known as one kind of most frequent lethal cancer in urological system. Recent literatures pointed out that its worse prognosis, frequent recurrence and more invasiveness caused increasing occurrence, presented charges for the treatment of RCC.^11^, ^12^ Besides, surgical resection is no longer applicable to recurrent cancer patients which prompted us to explore much more promising biomarkers for targeted therapeutic therapies. Herein, we conducted this work to confirm the function of ESCO2 in RCC carcinogenesis and figure out if it will provide secondary help for tumor diagnosis.

Sister chromatid cohesion is generated by acetylation of structural maintenance of chromosomes 3 (SMC3) mediated throughout the Eco1 family.^13^, ^14^ And human express two related acetyltransferase enzymes: Esco1 and Esco2. As mentioned in the literature review, Esco1 and Esco2 are essential for chromosomal stability and many human cancers are often formed from the instability of chromosomes.^15^, ^16^ Thus, increasing reports have been conducted to examine the action of ESCO1 and ESCO2 in various cancer carcinogenesis. For example, up‐regulated ESCO1 was found in bladder cancer,^17^ prostate cancer,^18^ and endometrial cancer,^19^ which was linked with the cellular behaviors. However, according to our analysis, ESCO1 was not a differentially expressional gene in RCC with none prognostic significance. Chen et al demonstrated that ESCO2 may be served as prognostic markers relying on its dysregulation in gastric cancer,^20^ lung squamous cell carcinoma,^21^ and melanomas.^22^ More interestingly, we gained the consistent results in RCC: through in vitro experiments, we found that ESCO2 knockdown significantly inhibited the proliferation, invasion and migration of RCC cells. These findings told us that the function of ESCO2 in cancers may be unified. Moreover, data from the TCGA dataset uncovered that the prevalence of RCC was usually accompanied by high expression of ESCO2. The shorter survival period and clinical pathological parameters had some certain connection with up‐regulation of ESCO2. Mechanistic exploration revealed that ESCO2 silencing was accompanied by a decrease in the expression level of PCNA, p53, and the AKT/mTOR pathway‐related proteins (p‐AKT and p‐mTOR). From these results, we can draw a conclusion that ESCO2 might play a positive role in the progression of RCC.

Prior studies have noted the importance of AKT/mTOR pathway in RCC evolution, further, cohesion‐regulated by ESCO2 was associated with the AKT/mTOR signaling.^23^, ^24^ In accordance with that, we conducted Western blot to assess whether the AKT/mTOR signaling pathway is involved in the regulation of ESCO2 in RCC progression and the results concluded our hypothesis. In addition, p53 has been reported as a major tumor suppressor, cooperating with the AKT/mTOR pathway.^25^, ^26^ The exertion of ESCO2 functions relied on the protein‐protein bindings, such as p53 and SMC3.^20^ Percival et al demonstrated that complete cohesion, abnormal chromosome segregation and genomic instability were induced by p53 and ESCO2 knockdown.^27^ Reduction of p53 in RCC cells transfected with si‐ESCO2 validated that there might have a correlation between ESCO2 and p53 in the development of RCC. Billon et al and Moldovan et al illustrated that PCNA can control the establishment of sister chromatid cohesion in S phase by Eco1‐mediated acetylation.^28^, ^29^ ESCO2 was overexpressed in S phase while disappeared in G2/M phase, indicating that ESCO2 may have specific function on the establishment of SCC during S phase in RBS cells.^30^ In our study, PCNA was also decreased after ESCO2 knockdown. However, one of the issues that emerged from these findings is the precise molecular mechanism of how ESCO2 manipulated the tumorigenesis with p53, PCNA, and the AKT/mTOR pathway is still not fully understood. This is an important issue, and then we should try our best to solve it in the future.

## 5 CONCLUSIONS

In conclusion, our data expounded that ESCO2 expression was significantly high‐regulated in RCC and correlated with the progression of RCC. Down‐regulation of ESCO2 attenuated cell proliferation, migration and invasion, which might be associated with the inactivation of the AKT/mTOR pathway. Additionally, the cancer‐related proteins, PCNA and p53, were also limited owing to the decline of ESCO2. These results enrich our comprehension of ESCO2's function and suggest that ESCO2 may be responsible for cancer therapeutic intervention by diagnosing RCC and forecasting prognosis.

## CONFLICT OF INTERESTS

The authors have no competing interests to declare.
